# CB2 Cannabinoid Receptor Targets Mitogenic Gi Protein–Cyclin D1 Axis in Osteoblasts

**DOI:** 10.1002/jbmr.228

**Published:** 2010-08-27

**Authors:** Orr Ofek, Malka Attar-Namdar, Vardit Kram, Mona Dvir-Ginzberg, Raphael Mechoulam, Andreas Zimmer, Baruch Frenkel, Esther Shohami, Itai Bab

**Affiliations:** 1 Bone Laboratory, Hebrew University of Jerusalem Jerusalem, Israel; 2 Institute of Dental Sciences, Hebrew University of Jerusalem Jerusalem, Israel; 3 Institute of Drug Research, Hebrew University of Jerusalem Jerusalem, Israel; 4 Institute of Molecular Psychiatry, University of Bonn Bonn, Germany; 5 Departments of Orthopedic Surgery and Biochemistry and Molecular Biology, Institute for Genetic Medicine, Keck School of Medicine, University of Southern California Los Angeles, CA, USA; 6 Department of Pharmacology and the David R Bloom Center for Pharmacy, Hebrew University of Jerusalem Jerusalem, Israel

**Keywords:** CB2 CANNABINOID RECEPTOR, CREB, CYCLIN D1, MAPKAPK2, MAP KINASE, MITOGENIC SIGNALING, OSTEOBLAST

## Abstract

CB2 is a Gi protein–coupled receptor activated by endo- and phytocannabinoids, thus inhibiting stimulated adenylyl cyclase activity. CB2 is expressed in bone cells and *Cb2* null mice show a marked age-related bone loss. CB2-specific agonists both attenuate and rescue ovariectomy-induced bone loss. Activation of CB2 stimulates osteoblast proliferation and bone marrow derived colony-forming units osteoblastic. Here we show that selective and nonselective CB2 agonists are mitogenic in MC3T3 E1 and newborn mouse calvarial osteoblastic cultures. The CB2 mitogenic signaling depends critically on the stimulation of Erk1/2 phosphorylation and de novo synthesis of *MAP kinase–activated protein kinase 2* (*Mapkapk2*) mRNA and protein. Further downstream, CB2 activation enhances CREB transcriptional activity and *cyclin D1* mRNA expression. The CB2-induced stimulation of CREB and cyclin D1 is inhibitable by pertussis toxin, the MEK-Erk1/2 inhibitors PD098059 and U0126, and *Mapkapk2* siRNA. These data demonstrate that in osteoblasts CB2 targets a Gi protein–cyclin D1 mitogenic axis. Erk1/2 phosphorylation and Mapkapk2 protein synthesis are critical intermediates in this axis. © 2011 American Society for Bone and Mineral Research.

## Introduction

CB2 is a seven-transmembrane domain receptor coupled to the inhibitory guanine nucleotide-binding regulatory protein subclass of G proteins. It is activated by endo- and phytocannabinoids, thus inhibiting stimulated adenylyl cyclase activity.(1,2) Unlike the neuronal CB1 cannabinoid receptor, CB2 is expressed predominantly in nonneuronal cells and has no psychoactivity. In bone, CB2 is expressed in osteoblasts, osteocytes, and osteoclasts. *Cb2* null mice show a marked age-related bone loss, and CB2-specific agonists both attenuate and rescue ovariectomy (OVX)–induced bone loss.(3–5) Notably, CB2 activation is mitogenic to osteoblasts in culture and increases the number of bone marrow colony-forming units osteoblastic.(3) At least one of the endocannabinoids present in bone, anadamide, stimulates osteoblast proliferation in vitro (data not shown).

In mammalian cells, the family of mitogen-activated protein (MAP) kinases provides a key link between membrane-bound receptors and changes in the pattern of gene expression. The MAP kinases are activated downstream of many different types of receptors, including tyrosine kinase receptors, cytokine receptors, and serpentine G protein–coupled receptors.(6,7) The MAP kinases consist of three subfamilies: the extracellular signal–regulated kinases 1 and 2 (Erk1/2), c-Jun N-terminal kinase/stress-activated kinase, and p38 MAP kinase. Further downstream, they regulate a multitude of transcription factors that control cell proliferation, survival, and differentiation.(8,9) In osteoblasts, Erk1/2-dependent phosphorylation cascades have been implicated in the regulation of proliferation and RUNX2 activity.(10–12) Activation of p38 has been demonstrated in osteoblasts undergoing differentiation after stimulation with bone morphogenetic protein 2 (BMP-2) and transforming growth factor β1 (TGF-β1).(13,14)

Downstream of Gi protein, CB2 regulates Erk1/2 and/or p38 phosphorylation. Depending on the cell type involved, this regulation is either stimulatory or inhibitory.(15–17) However, very little is known about CB2-triggered signaling events further downstream of these MAP kinases. Hence, in this study, we asked which of these MAP kinase subfamilies is used by CB2 in osteoblasts and what is the further downstream osteoblastic pathway that communicates CB2 mitogenic signals.

## Materials and Methods

### Materials

Polymethyl methacrylate Technovit 9100 was from Hareus Kulzer (Wehrheim, Germany). Calcein and pertussis toxin (PTX) were purchased from Sigma (cat. no. C-0875 and P-7208; St Louis, MO, USA). Tissue culture ingredients were from Biological Industries (Beit Haemek, Israel). Collagenase P was purchased from Roche Applied Science. Antibodies to phosphorylated and nonphosphorylated Erk1/2, p38 MAP kinase, and mitogen-activated protein kinase–activated protein kinase 2 (Mapkapk2) were from Cell Signaling Technologies (Danvers, MA, USA). The Erk1/2-activating kinase MEK inhibitors PD098059 and U0126 and p38 MAP kinase inhibitors SB203580 and SB202190 were from Calbiochem. Reagents for the luciferase assay were obtained from Promega (Madison, WI, USA). siRNA materials were from Santa Cruz Biotechnology (Santa Cruz, CA, USA). Colorimetric 5-Bromo-2-Deoxyuridine (BrdU) Labeling and Detection Kit III was from Roche Diagnostics. Reagents for real-time RT-PCR were from Applied Biosystems (Foster City, CA, USA). AM-1241 was from Alexis Biochemicals. The EZ-ChIP kit was purchased from Upstate Millipore (Billerica, MA, USA), and anti-phospho-CREB (on serine 133) antibody was from Cell Signaling Technologies. The Qiaquick spin kit for DNA extraction was from Qiagen.

### Animals

Wild-type (WT) C57Bl/6J mice were from Harlan Israel. *Cb2* null mice(18) were crossed for 10 generations to WT C57BL/6J mice to generate a congenic C57BL/6J *Cb2*^–/–^ strain and further bred under SPF conditions at the Hebrew University Ein Kerem Animal Facility. The effect of CB2 signaling in OVX animals was analyzed in normal C57Bl/6 mice. Animals were injected intraperitoneally with HU-308 at 20 mg/kg per day for 6 weeks, beginning 6 weeks after OVX. The injection vehicle was ethanol/emulphor/saline (1:1:18). Sham OVX surgery comprised exposure of the ovaries. To study bone formation, newly formed bone was vitally labeled with the fluorochrome calcein injected intraperitoneally (15 mg/kg) 4 days and 1 day before euthanization. At euthanization, the femurs were separated, fixed for 24 to 48 hours in phosphate-buffered formalin, and further kept in 70% ethanol. The experimental protocols were approved by the Institutional Animal Care and Use Committee of the Hebrew University of Jerusalem.

### Histomorphometry

Femurs were embedded undecalcified in polymethyl methacrylate. Longitudinal sections through the midfrontal plane were left unstained for dynamic histomorphometry based on vital calcein double labeling.(19)

### Cell cultures

MC3T3 E1 osteoblastic cells were maintained as reported previously.(20) Cells were incubated for 5 to 6 days in osteogenic medium until subconfluence to allow for increased CB2 expression.(3) Newborn mouse calvarial osteoblasts (NeMCOs) were prepared from 5-day-old WT mice and *Cb2* null mice by successive collagenase digestions.(21)

### Western analysis

MC3T3 E1 cells were seeded in 10-cm dishes at 5 × 10^5^ cells/dish and incubated in osteogenic medium. Subconfluent cultures were serum-starved overnight in 0.5% bovine serum albumin containing α-MEM. Thereafter, the cells were incubated for various time periods ranging from 5 minutes to 2 hours in the same medium with or without cannabinoid ligands and MAP kinase inhibitors. The cells then were rinsed with cold phosphate-buffered saline and lysed with 50 mM of Tris-HCl buffer (pH 7.5), 1% Triton X-100, 150 mM of NaCl, 1 mM of EGTA, 50 mM of β-glycerophosphate, 1 mM of NaF, 10 µg/mL of leupeptin, 10 µg/mL of aprotinin, 0.5 mM of phenylmethylsulfonyl fluoride, and 1 mM of sodium orthovanadate. The cells then were scraped off using a rubber policeman, and the lysates were clarified by centrifugation at 12,000*g* for 15 minutes. Samples from each lysate, containing 40 to 120 µg of protein, were fractionated by SDS-PAGE and then electroblotted onto nitrocellulose membranes. The membranes were blocked with nonfat dry milk in TRIS-buffered saline/Tween-20 solution. The Western blots were probed with antibodies to phosphorylated Erk1/2, Erk1/2, phosphorylated p38, p38, phosphorylated Mapkapk2 (Thr222), and Mapkapk2. Proteins on the Western blots were detected using the EZ-ECL chemiluminescent detection system (Biological Industries).

### DNA synthesis measurement

After serum starvation, the cells were incubated for 24 hours in 0.5% bovine serum albumin with or without the ligands and with or without PTX, inhibitors of MAP kinase phosphorylation, and Mapkapk2 siRNA. This was followed by labeling with BrdU for 24 hours and determination of its incorporation into DNA using a commercial kit according to the manufacturer's instructions.

### RNA interference

Inhibition of Mapkapk2 expression was achieved using a commercial siRNA kit according to the manufacturer's instructions. The kit included mouse Mapkapk2 siRNA (cat. no. SC-35856), control siRNA (cat. no. SC-37007), siRNA dilution buffer (cat. no. SC-29527), siRNA transfection reagent (cat. no. SC-29528), and siRNA transfection medium (cat. no. SC-36868). Briefly, MC3T3 E1 cells were seeded in 96-well plates, 5 × 10^3^ cells/well in 200 µL of antibiotic-free medium. Cultures at approximately 50% confluence were transfected with control or Mapkapk2 siRNA in transfection medium containing transfection reagent. After 5 hours of incubation in control and Mapkapk2 siRNA, the cells were serum-starved for 2 hours and then challenged with HU-308.

### Luciferase assay

MC3T3 E1 cells stably transfected with a luciferase construct reporting on CREB transcriptional activity and containing three copies of a canonical CRE (pCRE3-luc) were reported previously.(12) To test the effect of HU-308 on CREB transcriptional activity, the stably transfected cells, heretofore MC3T3 E1/CRE-luc, were plated in 48-well plates and grown for 48 hours in α-MEM supplemented with 10% fetal calf serum. After 2 hours of starvation, the cells were fed with HU-308 with or without PD098059 in α-MEM containing 0.5% bovine serum albumin. The cells were harvested 16 hours thereafter and lysed in “reporter lysis buffer” (Promega). Luciferase activity was determined using a microtiter plate luminometer (LB940 Multilabel Reader, Berthold Technologies).

### Real-time RT-PCR

Total RNA was isolated from MC3T3 E1 and NeMCO cells incubated for 4 hours with or without HU-308 and MAP kinase inhibitors using the TRI Reagent Kit (Molecular Research Center, Inc., Cincinnati, OH, USA) followed by a phenol-chloroform phase extraction and isopropyl precipitation. RNA quality was assessed by light absorbance at 260 and 280 nm and by agarose gel electrophoresis and ethidium bromide staining. Real-time RT-PCR analysis for *Mapkapk2* and *cyclin D1* mRNA levels was carried out by using Applied Biosystems Taqman Gene Expression Assays (respective assay IDs Mm01288465_m1 and Mm00432359_m1). Data were normalized to *β-actin* (assay ID Mm00607939_s1). Relative quantity (RQ) data were calculated as percent maximal expression.

### Chromatin immunoprecipitation (ChIP**)**

Chromatin immunoprecipitation assays were carried out 5 hours after a challenge with 10^−8^ M HU-308 or vehicle according to the manufacturer's instructions. DNA was extracted and post-ChIP RT-PCR carried out using a phospho-CREB antibody. Gels were subjected to computerized densitometry, with negative IgG controls being subtracted, and the values were normalized using internal control for RNA polymerase II immunoprecipitations on the promoter of *GAPDH*. Primer sequences for *GAPDH* were forward, TAC TAG CGG TTT TAC GGG CG; reverse, TCG AAC AGG AGC AGA GAG CGA. The primer sequences for *cyclin D1* CRE promoter element were forward, TCC CAG TTT GGA GAG AAG CA; reverse, AGA GAT CAA AGC CGG GCA GAG AAA.

### Statistical analysis

Analysis of variance was employed for statistical analysis. When significant differences were indicated by analysis of variance, group means were compared using the Student-Newman-Keuls test for pairwise comparisons.

## Results

We have reported previously that the CB2-specific agonist HU-308(22) stimulates endosteal bone formation and attenuates OVX-induced bone loss. In this model, HU-308 did not induce an increase in trabecular bone formation apparently because it was already enhanced as part of the high bone turnover triggered by OVX.(3) However, a micro–computed tomographic (µCT) analysis showed that daily administration of HU-308 also can rescue OVX-induced trabecular bone loss.(4) Because in cell cultures CB2 activation is mitogenic to osteoblasts, and because osteoblast number is the major determinant of bone formation,(21) we assessed whether CB2 activation affects trabecular bone formation in the more permissible rescue assay. Indeed, we show here in the same femoral specimens that HU-308 stimulates trabecular and endosteal bone formation (Supplemental Fig. S1), providing a rationale for the in-depth analysis of the CB2 mitogenic mechanism in osteoblasts.

**Fig. 1:**
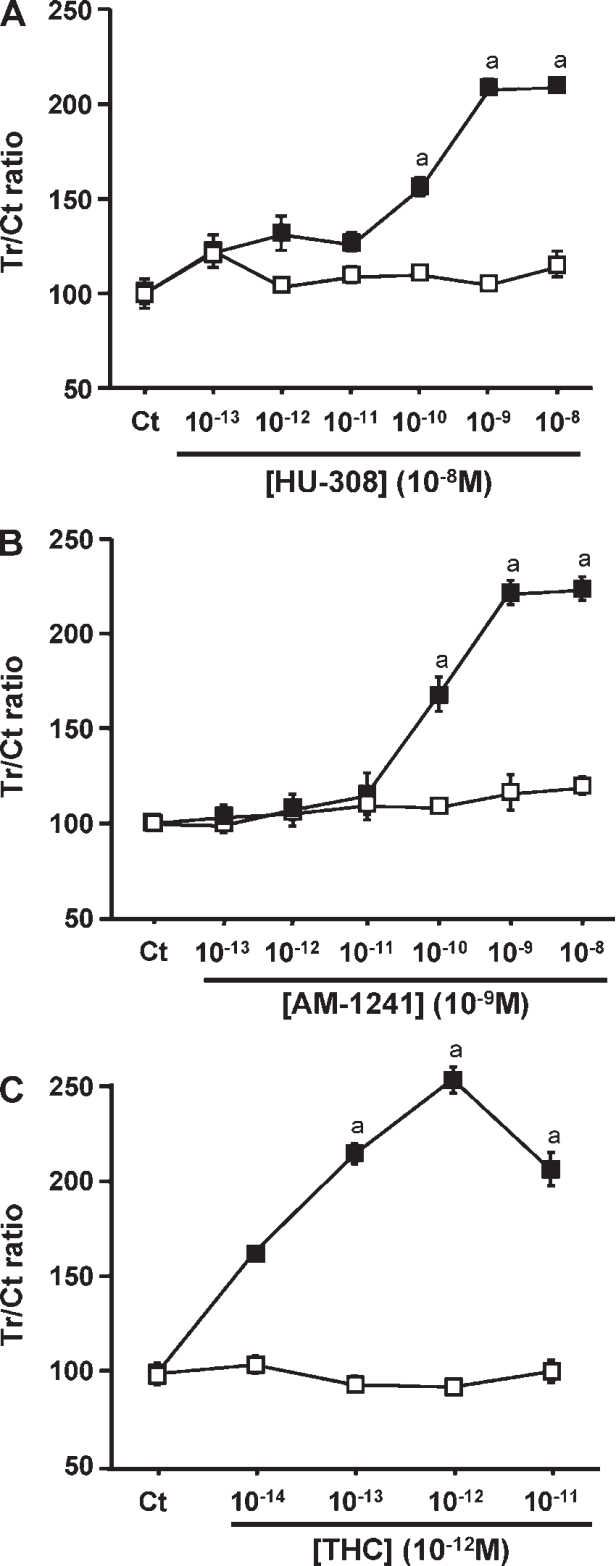
CB2 agonists stimulate osteoblast mitogenesis. BrdU uptake by NeMCOs from wild-type (*full squares*) and *Cb2^−/−–^* mice (*open squares*). (*A*) HU-308. (*B*) AM-1241. (*C*) THC. Data are mean ± SE of Treated/Control ratio obtained in 5 culture wells per condition. Tr = agonist-treated cultures; Ct = agonist-free control cultures. ^a^*p*< .05 versus Ct.

To confirm that the mitogenic activity of HU-308 is shared by other CB2 agonists, we compared its effect on DNA synthesis with that of another specific CB2 agonist, AM-1241,(23) and the CB1/CB2 agonist Δ^9^-tetrahydrocannabinol (THC).(24) As in bone marrow–derived and MC3T3 E1 osteoblasts,(3) HU-308 stimulated BrdU incorporation into NeMCO DNA dose-dependently, with a maximal, greater than 2-fold effect at 10^−9^ to 10^−8^ M (Fig. 1*A*). Analyzing AM-1241 in the same system demonstrated a similar dose-response relationship (Fig. 1*B*). THC was also mitogenic to osteoblasts, but it was markedly more potent than the other agonists, having its peak effect at 10^−12^ M (Fig. 1*C*). Importantly, none of the agonists was mitogenic in NeMCO derived from CB2-deficient mice. Taken together, these data confirm the mitogenic activity of CB2, which is independent of the type of agonist used.

**Fig. 2:**
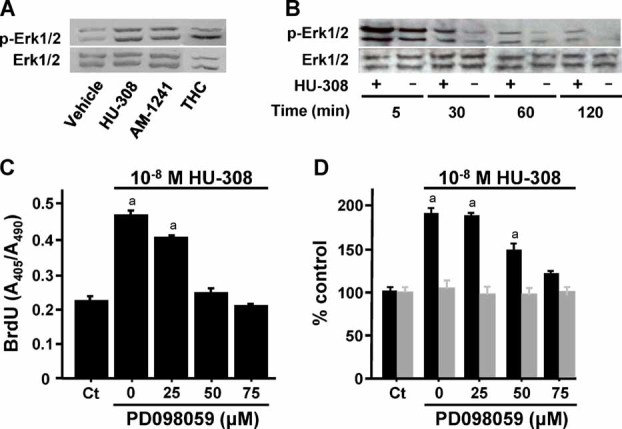
CB2 mitogenic signaling in osteoblastic cells is mediated by Erk1/2. (*A*,*B*) Stimulation of Erk1/2 phosphorylation in MC3T3 E1 cells: (*A*) 2-hour challenge with selective CB2 agonists HU-308 and AM-1241 and CB1/CB2 agonist THC at 10^−8^, 10^−9^, and 10^−12^ M, respectively; (*B*) 5 to 120 minute challenge with 10^−8^ M HU-308. (*C*,*D*) Inhibition of HU-308–stimulated DNA synthesis by MEK-Erk1/2 inhibitor PD098059: (*C*) MC3T3 E1 cells; (*D*) NeMCO cultures derived from wild-type (*black bars*) and *Cb2^–/–^* animals (*gray bars*). Data are mean ± SE obtained in three culture wells per condition. ^a^*p* < .05 versus Ct.

Mitogenic signals in many cell types, including osteoblasts, are mediated by MAP kinases regardless of the receptor class involved, for example, tyrosine kinase or G protein–coupled receptors.(6,7) Furthermore, MAP kinases are also activated by CB2-triggered signals.(15) Hence, we tested whether in osteoblasts too, CB2 agonists stimulate MAP kinase phosphorylation. Indeed, we show that prolonged challenge of MC3T3 E1 osteoblasts with optimal doses of either HU-308, AM-1241, or THC stimulated Erk1/2 phosphorylation (Fig. 2*A*). A detailed temporal analysis demonstrated that the enhancement of Erk1/2 stimulation persists at least between 5 minutes and 2 hours from the time of exposure to HU-308 (Fig. 2*B*), suggesting that receptor desensitization is very slow, if any. The HU-308 stimulation of BrdU incorporation into newly synthesized DNA in MC3T3 E1 and WT NeMCO cells and the HU-308-induced increase in the number of these cells were dose-dependently suppressed by the MEK-Erk1/2 pathway inhibitors PD098059 and U0126 (Figs. 2*C, D*, and Supplemental Fig. S2*A*). Consistent with the BrdU data (Fig. 1), CB2-deficient NeMCOs were nonresponsive in this experimental setting (Fig. 2*D*). These results suggest that stimulation of the Erk1/2 pathway is required for CB2 mitogenic signaling.

**Fig. 3:**
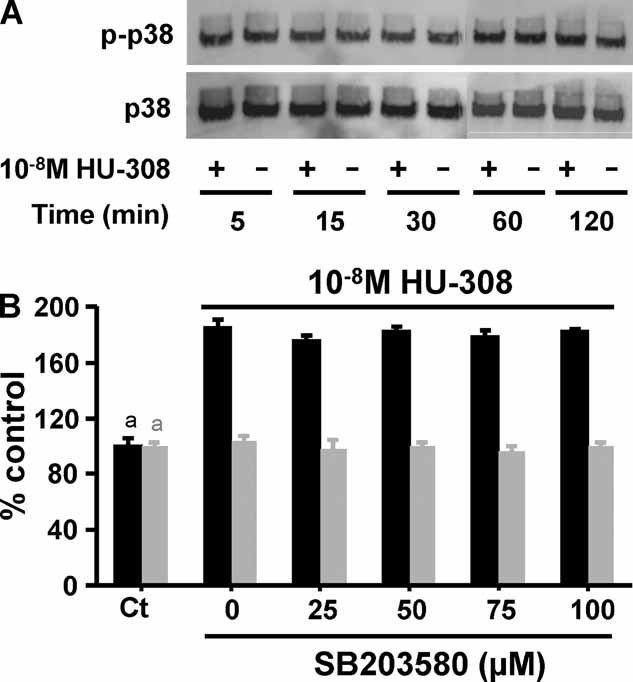
p38 is not involved in CB2 signaling in osteoblasts. (*A*) MC3T3 E1 osteoblastic cells were incubated with or without CB2-specific agonist HU-308. Western blot analysis was performed with anti-phosphorylated p38 (p-p38) or anti-p38 antibodies. (*B*) Cell counts in NeMCO cultures derived from wild-type (*black bars*) and *Cb2^–/–^* animals (*gray bars*) incubated for 48 hours with or without HU-308 and the indicated doses of p38 phosphorylation inhibitor SB203580. Data are mean ± SE obtained in three culture wells per condition. ^a^*p* < .05.

To rule out the possible involvement of p38 in the CB2-trigered mitogenic signaling cascade, we analyzed p38 activation employing a similar approach. HU-308 did not affect p38 phosphorylation in MC3T3 E1 osteoblasts (Fig. 3*A*). In addition, SB203580 and SB202190, specific inhibitors of p38,(25,26) did not inhibit the HU-308 stimulation of DNA synthesis and cell number in WT NeMCO even at 100 and 25 µM, respectively (Fig. 3*B* and Supplemental Figure S2*B*). As in the experiment with PD098059 (Fig. 2*D*), *Cb2* null cells did not respond to either HU-308 or inhibition of p38 (Fig. 3*B*).

**Fig. 4:**
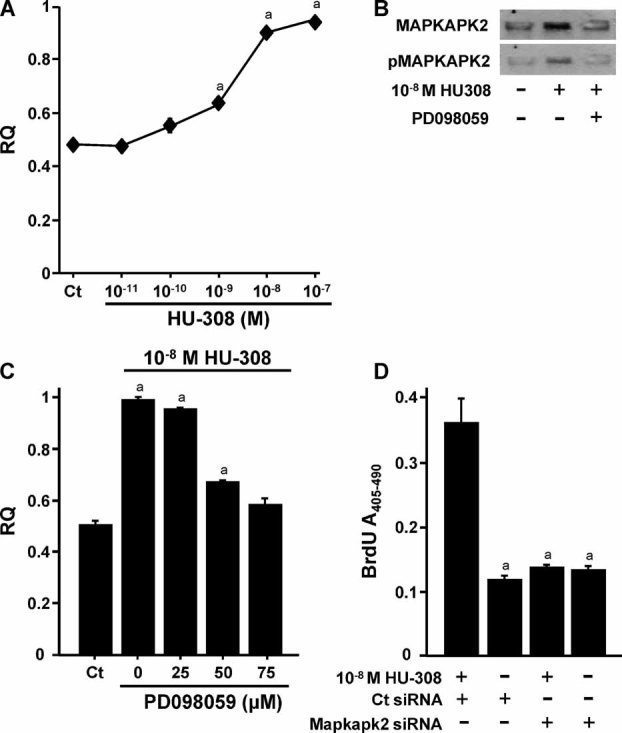
Stimulation of *Mapkapk2* mRNA and protein downstream of Erk1/2 is essential for CB2 mitogenic signaling in osteoblasts. (*A–C*) MC3T3 E1 cells were challenged with HU-308 for 8 hours: (*A*) dose-response analysis of *Mapkapk2* mRNA levels analyzed by real-time PCR; (*B*) Western blot analysis with antibodies against Mapkapk2 or phosphorylated Mapkapk2; (*C*) Cells were incubated with HU-308 with or without the MEK-Erk1/2 inhibitor PD098059. (*D*) Inhibition of CB2 mitogenic signaling in MC3T3 E1 cells by siRNA to Mapkapk2. Quantitative data are mean ± SE obtained in six culture dishes per condition. ^a^*p* < .05 versus Ct or cultures treated with HU-308 and Ct siRNA.

In a previous study on Gi protein–mediated mitogenic effect in osteoblasts, we unraveled a signaling pathway downstream of Erk1/2 that consists of elevated *Mapkapk2* mRNA levels and stimulation of CREB.(12) Because of the similarity between the upstream players in the earlier and this study, that is, Gi protein and Erk1/2 stimulation, we set out to assess the use of these downstream events by CB2. Expectedly, in MC3T3 E1 cells, an 8-hour challenge with HU-308 stimulated *Mapkapk2* mRNA levels dose-dependently at 10^−9^ to 10^−7^ M (Fig. 4*A*). The upregulation of *Mapkapk2* mRNA triggered by CB2 activation resulted in a parallel increase at the protein level (Fig. 4*B*). We also investigated whether the increase in the Mapkapk2 protein was associated with an alteration in its phosphorylation state. Western analyses with antibodies against phosphorylated Mapkapk2 yielded essentially the same results as those obtained with the pan antibody, suggesting that the HU-308-induced increase in Mapkapk2 protein was not associated with alterations in its phosphorylation status (Fig. 4*B*). The increases in *Mapkapk2* mRNA levels were blocked by PD098059 and U0126 (Fig. 4*C* and Supplemental Fig. S2*C*), indicating that activation of the MEK-Erk1/2 pathway is critical for mediating the stimulation of Mapkapk2 expression induced by CB2 activation. We used RNA interference to silence the *Mapkapk2* gene and determine whether Mapkapk2 plays a role in CB2 mitogenic signaling. Compared with control siRNA, *Mapkapk2* siRNA mitigated the stimulatory effect of HU-308 on DNA synthesis (Fig. 4*D*), indicating that synthesis of Mapkapk2 is necessary for the mitogenic signaling of CB2.

**Fig. 5:**
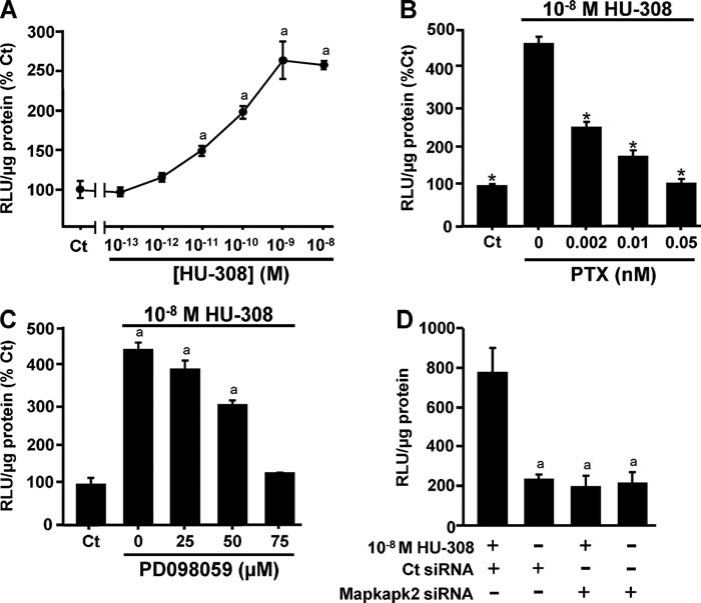
CB2 mitogenic signaling in osteoblasts involves stimulation of CREB transcriptional activity downstream of enhanced de novo *Mapkapk2* mRNA levels. MC3T3 E1/CRE-luc cells were challenged with HU-308 for 8 hours (*A–C*) or 5 hours (*D*), and optical density was measured as luciferase relative light units (RLUs). (*A*) Dose-response analysis. (*B–D*) Arrest of CB2-stimulated CREB activity by: (*B*) selective Gi protein inhibitor pertusis toxin; (*C*) MEK-Erk1/2 inhibitor PD098059; (*D*) *Mapkapk2* siRNA. Data are mean ± SE obtained in six culture dishes per condition. ^a^*p* < .05 versus Ct or cultures treated with HU-308 and Ct siRNA.

Because in osteoblasts and other cells CREB is one of the main targets of Mapkapk2,(12,27) we assessed the effect of HU-308 on its transcriptional activity. The effect of HU-308 was measured in MC3T3 E1/CRE-luc cells, which are stably transfected with a luciferase construct that reports on CREB transcriptional activity. Luciferase assays then were performed to functionally investigate the transcriptional outcome after stimulation of the Gi protein–Erk1/2–Mapkapk2 cascade. As shown in Fig. 5*A*, CB2 activation in the MC3T3 E1/CRE-luc cells stimulated luciferase activity dose-dependently, peaking at 10^−9^ to 10^−8^ M. The stimulation of CREB transcriptional activity by CB2 activation was mitigated dose-dependently by PTX (Fig. 5*B*), by the MEK-Erk1/2 inhibitors PD098059 and U0126 (Fig. 5*C* and Supplemental Fig. S2*C*), and by *Mapkapk2* siRNA (Fig. 5*D*). Jointly, the established relationship between Mapkapk2 and CREB, the CB2-induced activation of both proteins, and the attenuation of the CB2-mediated CREB transcriptional activity by PTX, MEK/Erk1/2 inhibitors PD098059 and U0126, and *Mapkapk2* siRNA (Fig. 5 and Supplemental Fig. S2) suggests that CREB is the downstream link in a CB2-activated mitogenic signaling axis that depends on the stimulation of Erk1/2 activity and Mapkapk2 protein synthesis.

**Fig. 6:**
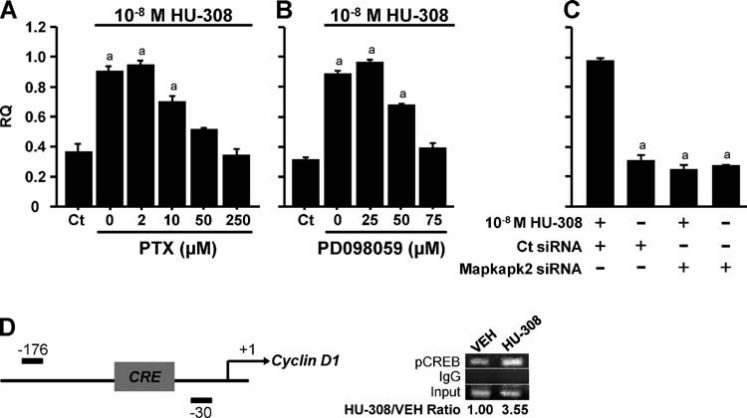
CB2 mitogenic signaling in osteoblasts involves stimulation of *cyclin D1* mRNA expression. MC3T3 E1 cells were incubated with HU-308 with or without the following inhibitors: (*A*) pertussis toxin (PTX), (*B*) MEK-Erk1/2 inhibitor PD098059, or (*C*) *Mapkapk2* siRNA. (*D*) Chromatin immunoprecipitation analysis assay of phospho-CREB (pCREB) binding to CRE in cyclin D1 promoter. Data in panels *A* to *C* are mean ± SE obtained in six culture dishes per condition. ^a^*p* < .05 versus Ct.

Several G protein–coupled receptors (GPCRs) control osteoblast proliferation through the regulation of *cyclin D1* expression by CREB.(28) Therefore, we assessed the effect of CB2 activation on *cyclin D1* mRNA. Indeed, HU-308 stimulates osteoblastic *cyclin D1* mRNA levels, and this enhancement is inhibitable by PTX, PD098059, U0126, and *Mapkapk2* siRNA (Fig. 6*A–C* and Supplemental Fig. S2*D*), suggesting that cyclin D1 is a mitogenic regulator targeted by CB2. In line with these findings, HU-308 enhanced the binding of phospho-CREB to the promoter of cyclin D1 (Fig. 6*D*).

## Discussion

**Fig. 7:**
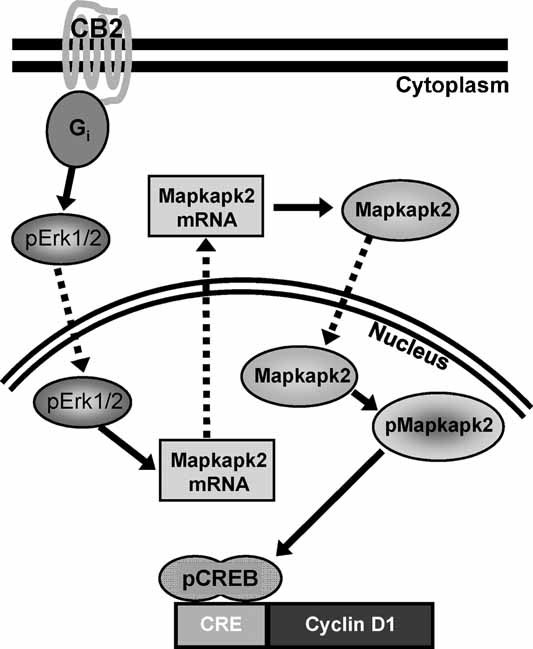
Model of CB2 mitogenic signaling in osteoblasts. Gi protein activation is followed by enhanced ERK1/2 phosphorylation and accumulation of Mapkapk2. p-Mapkapk2 phosphorylates and activates CREB, resulting in transcriptional activation of cyclin D1 and cell proliferation. *Arrows* = activation events; *dashed arrows* = translocation events.

In this study we show that downstream of Gi protein, CB2 mitogenic signaling in osteoblasts involves phosphorylation of Erk1/2 and de novo *Mapkapk2* mRNA and protein synthesis. Further downstream, CB2 activation stimulates CREB transcriptional activity and cyclin D1 expression (Fig. 7), both inhibitable by suppressing Erk1/2 activation and Mapkapk2 protein synthesis.

Using the WT- and *Cb2*^*–/–*^-derived NeMCO model and the selective CB2 agonists HU-308 and AM-1241,(22,23) we confirm here that CB2 activation is mitogenic to osteoblasts. By comparison with these agonists, the mitogenic effect of the CB1/CB2 agonist THC(29) is markedly more potent. In osteoblasts, CB1 is expressed at very low levels.(3,30,31) Regardless of the CB1 level, the failure of THC to enhance DNA synthesis in NeMCOs derived from *Cb2* null mice suggests that osteoblastic CB1 is not involved in proliferative activity.

Based on the reported CB2 signaling in a variety of cell types, and because ERK1/2 and/or p38 mediate a handful of extracellular signals in osteoblasts,(27,32,33) we tested whether the CB2 agonists increase the phosphorylation of these kinases. Indeed, all three agonists potently stimulated Erk1/2 phosphorylation. Moreover, this stimulation and the CB2 mitogenic action were blocked by the specific Erk1/2 inhibitors PD098059 and U0126, indicating that ERK1/2 activation is a critical link in the CB2-triggered stimulation of DNA synthesis. Cellular responses mediated by GPCRs are usually rapidly attenuated, a process termed *desensitization*. By contrast, the prolonged activation of osteoblastic Erk1/2 by HU-308 suggests that in osteoblasts the CB2-induced Gi protein activation is attenuated very slowly. The rate of desensitization is regulated by phosphorylation of G protein–coupled receptor kinases (GRKs), which, in turn, promote the binding of arrestins to the receptor, resulting in the uncoupling of GPCRs from G proteins.(34,35) Hence, these findings further suggest a specific slow-acting set of GRKs and arrestins in use of osteoblastic CB2. Unlike Erk1/2, p38 is not stimulated by CB2 activation, and the p38 inhibitors SB203580 and SB202190 do not affect the CB2-mediated increase in cell number. These data strongly suggest that Erk1/2 are the only MAP kinases involved in the CB2 mitogenic signaling.

We have noted previously that unlike the case of many other mitogens such as platelet-derived growth factor and epidermal growth factor, whereby stimulation of DNA synthesis is measurable already after 24 hours,(36,37) approximately 48 hours are required before the mitogenic action of CB2 becomes traceable.(3) We therefore assumed that the CB2-activated signaling cascade downstream of Erk1/2 involves de novo mRNA and protein syntheses. A likely candidate for such a signaling event was Mapkapk2, whose involvement in a delayed Gi protein–mediated mitogenic signaling had been reported.(12) Indeed, we demonstrate that CB2 induces accumulation of *Mapkapk2* mRNA and protein and that these events are critical in CB2 mitogenic signaling. We further show that stimulation of the nonphosphorylated Mapkapk2 substrate is associated with a parallel stimulation of the phosphorylated Mapkapk2 product. The increase in activated Mapkapk2 is traceable after a several-hour challenge with a selective CB2 agonist, in line with the requirement for de novo protein synthesis. That Mapkapk2 protein synthesis is essential for the CB2 mitogenic activity is demonstrated by its inhibition using *Mapkapk2* siRNA. Although Mapkapk2, also designated MK2,(38) is generally accepted as a substrate for p38,(39,40) it was identified originally as an Erk target.(41) Furthermore, PD098059 partially inhibits the activation of Mapkapk2 by the GPCR agonist endothelin 1.(42) Although it is not clear to what extent the Erk stimulation of Mapkapk2 depends on de novo protein synthesis, it is suggested by several instances where prolonged challenging was required for its activation.(43,44) That the CB2 stimulation of Mapkapk2 synthesis and phosphorylation depends on Erk1/2 activation is shown by its complete arrest with PD098059 and U0126.

CREB is one of the best characterized stimulus-induced transcription factors. It activates transcription of target genes in response to a diverse array of stimuli, including peptide hormones and growth factors that activate a variety of protein kinases.(45) In osteoblastic cells, CREB is activated by extracellular stimuli, including parathyroid hormone (PTH), epidermal growth factor, and prostaglandin E_2_.(13,46,47) More relevant to this study is the Gi protein–Erk1/2-Mapkapk2-CREB mitogenic pathway triggered by the osteogenic growth peptide.(12) We show here that this pathway communicates to the nucleus signals elicited by CB2 agonists. The cyclin D1 promoter has a CRE consensus sequence, rendering this transcription factor a major target for GPCR-CREB mitogenic signaling.(48) CREB-induced upregulation of cyclin D1 has been implicated recently in the PTH mitogenic effects in osteoblasts.(49) Downregulation of CREB transcriptional activity has been portrayed in the restrain of osteoblast proliferation and bone formation that follows activation of the Htr1b serotonin receptor.(28) The inverse activity of these GPCRs, including the present CB2 stimulation of Mapkapk2, CREB, cyclin D1, and DNA synthesis, suggests that the Gi protein–cyclin D1 axis transmits dose-related mitogenic signals in osteoblasts.

This study confirms that Erk1/2 phosphorylation and Mapkapk2 protein synthesis are critical for the Gi protein–cyclin D1 mitogenic axis in osteoblasts. These results in osteoblastic cells suggest further studies to assess the in vivo significance of this axis in the skeleton and other tissues alike.
