# Supplementary material for: CB2 Cannabinoid Receptor Targets Mitogenic Gi Protein–Cyclin D1 Axis in Osteoblasts
Source: J Bone Miner Res. 2010 Aug 27;26(2):308–16. doi: 10.1002/jbmr.228 (PMC3179350; doi:10.1002/jbmr.228)
Supplement: Supplementary file 1 [file jbmr0026-0308-SD1.ppt]

## Slide 1
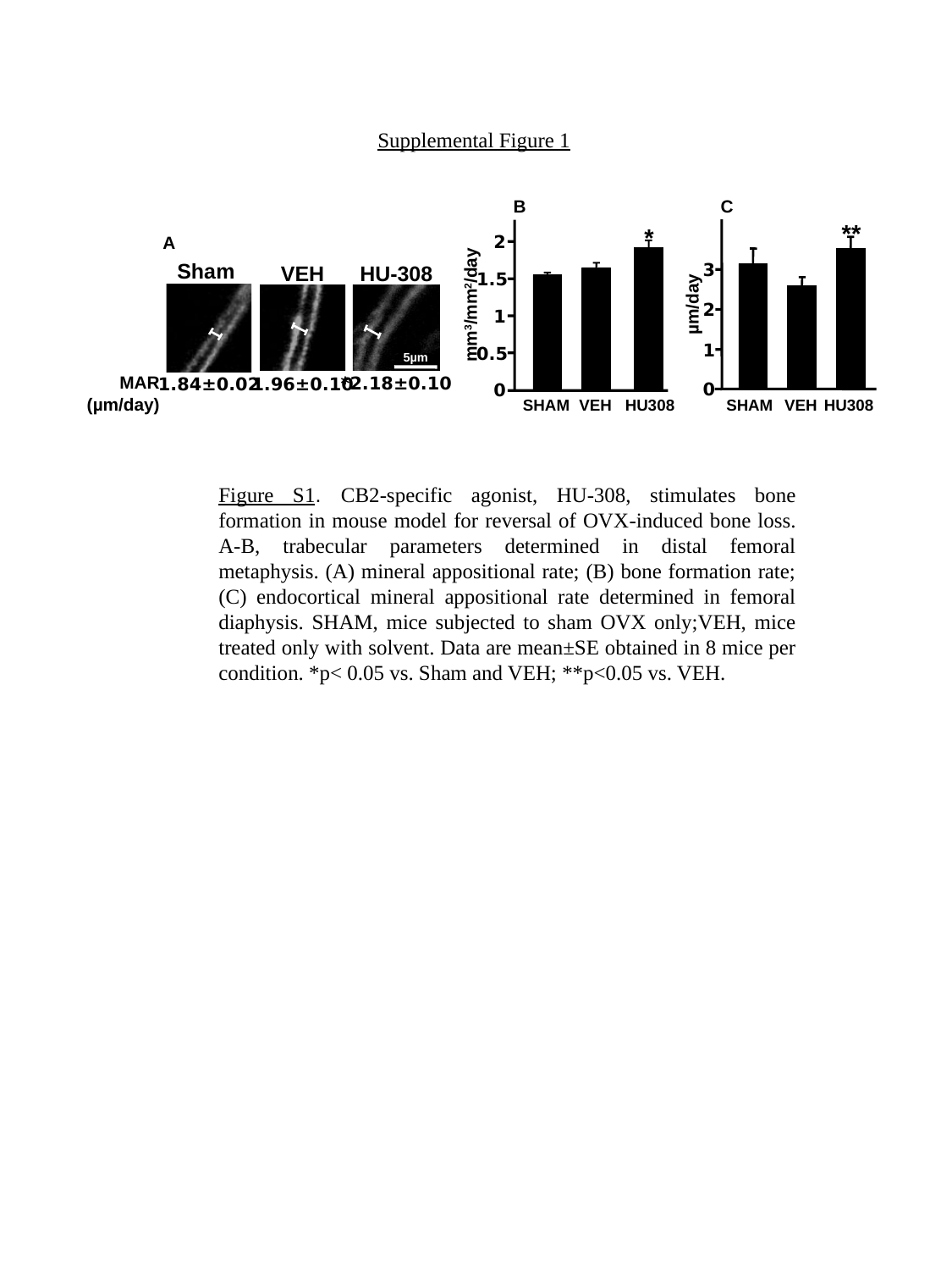

Supplemental Figure 1
B
C
**
*
A
2
Sham
VEH
HU-308
3
1.5
mm3/mm2/day
µm/day
2
1
1
0.5
5µm
MAR (µm/day)
2.18±0.10*
1.84±0.02
1.96±0.10
0
0
SHAM
VEH
HU308
SHAM
VEH
HU308
Figure S1. CB2-specific agonist, HU-308, stimulates bone formation in mouse model for reversal of OVX-induced bone loss. A-B, trabecular parameters determined in distal femoral metaphysis. (A) mineral appositional rate; (B) bone formation rate; (C) endocortical mineral appositional rate determined in femoral diaphysis. SHAM, mice subjected to sham OVX only;VEH, mice treated only with solvent. Data are mean±SE obtained in 8 mice per condition. *p< 0.05 vs. Sham and VEH; **p<0.05 vs. VEH.

## Slide 2
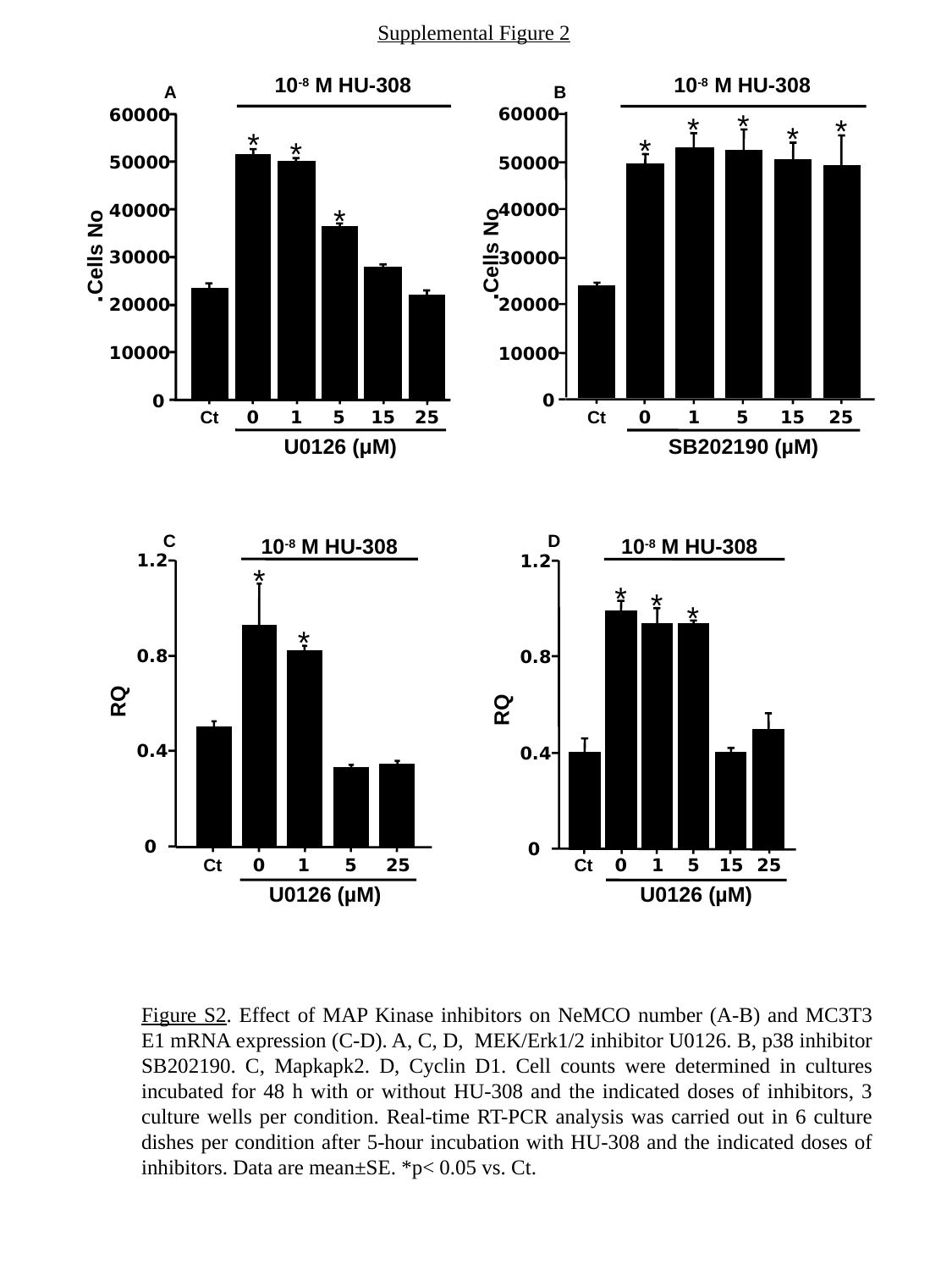

Supplemental Figure 2
10-8 M HU-308
10-8 M HU-308
A
B
*
*
60000
60000
*
*
*
*
*
50000
50000
*
40000
40000
Cells No.
Cells No.
30000
30000
20000
20000
10000
10000
0
0
Ct
0
1
5
15
25
Ct
0
1
5
15
25
U0126 (μM)
SB202190 (µM)
C
D
10-8 M HU-308
10-8 M HU-308
1.2
1.2
*
*
*
*
*
0.8
0.8
RQ
RQ
0.4
0.4
0
0
Ct
0
1
5
25
Ct
0
1
5
15
25
U0126 (µM)
U0126 (µM)
Figure S2. Effect of MAP Kinase inhibitors on NeMCO number (A-B) and MC3T3 E1 mRNA expression (C-D). A, C, D, MEK/Erk1/2 inhibitor U0126. B, p38 inhibitor SB202190. C, Mapkapk2. D, Cyclin D1. Cell counts were determined in cultures incubated for 48 h with or without HU-308 and the indicated doses of inhibitors, 3 culture wells per condition. Real-time RT-PCR analysis was carried out in 6 culture dishes per condition after 5-hour incubation with HU-308 and the indicated doses of inhibitors. Data are mean±SE. *p< 0.05 vs. Ct.
